# Comparison of Temperature Changes Between Holmium-YAG Laser and Thulium Fiber Laser in an In Vitro Setting

**DOI:** 10.5152/tud.2025.24100

**Published:** 2025-01-03

**Authors:** Vaibhavkumar Patel, Kshitij Raghuvanshi, Krushnadevsinh Jadeja, Rajeev Chaudhari

**Affiliations:** Department of Urology, Ruby Hall Clinic, Pune, India

**Keywords:** Holmium laser, Thulium fiber laser, temperature assessment, irrigation fluid, laser lithotripsy

## Abstract

**Objective:**

We aimed to evaluate and compare the rise in the temperature for the safety of the ureter and kidney parenchyma when firing the holmium laser and the thulium fiber laser (TFL).

**Methods:**

We performed a laboratory experiment to measure the rise in temperature upon firing holmium laser and a TFL in a 10 cm^3^ transparent test tube in an outdoor environment and then in a container with normal saline.

**Results:**

In a 10 cc test tube with static water at 25°C, the rise in temperature with holmium and TFL depends on the firing time, keeping power constant. On continuous firing for 10, 20, and 30 seconds, the temperature rose to 35.1, 42.7, and 53.2°C with holmium. The temperature went up to 38.3, 46.8, and 55.4°C when TFL was used. The power was kept at 10 W for both types of lasers. The temperature rise was relatively low when the test tube was immersed in a water container. It was even lower with irrigation.

**Conclusion:**

The rise in the temperature reaches to hazardous level in static water with a firing time of 30 seconds, which is almost equivalent to holmium and TFL. Thus, while using lasers in RIRS and ureteroscopy, the firing time should not exceed 20 seconds and adequate irrigation is required to prevent damage to the tissues. Also, the rise in temperature was almost equivalent to holmium and TFL; hence, TFL can be safely used in laser lithotripsy in any part of the genitourinary (GU) system.

**Main Points**

- Lasing time is one of the most important factors for temperature rise with both holmium and TFL.

- The rise in temperature is equal to TFL and holmium when fired for the same duration.
- There is a necessity to incorporate safety mechanisms to prevent over-firing, which can lead to thermal injury to the tissue and become a factor in ureteral stricture.

## Introduction

Endoscopic surgery has become the mainstay for the management of urolithiasis. Various modalities like electrohydraulic (EHL), pneumatic lithotripsy (PL), and ultrasonic lithotripsy (UL) were major advancements in the field of endourology. Electrohydraulic was good for bladder stones but was too strong for ureteric stones. Pneumatic lithotripsy and UL were very efficient, cost-effective, and safe. But, as they use a rigid probe, their use was limited to the rigid and semi-rigid scope, and miniaturization of the instrument became difficult.^1^ Pneumatic lithotripsy also caused retropulsion, which became a major disadvantage in upper ureteric stones and hydroureter.

The advent of lasers has overcome these limitations. The advancement in laser technology has elevated the capabilities of ureteroscopy (URS) to new heights.^2,3^ The small size of laser fibers and ease of deflection have led to the development of smaller diameter semirigid ureteroscopes and flexible ureteroscopes. It also took care of retropulsion to a very large extent.

In a span of less than 10 years, the holmium:yttrium-aluminum-garnet laser (Ho:YAG) became the gold standard for endoscopic laser lithotripsy, and it has maintained its supremacy for the past two decades.^4^ Recently, thulium laser fiber has been introduced, and several aspects, such as its effectiveness in dusting and fragmenting, stone ablation thresholds and rates, the formation of gas bubbles, and the assessment of collateral damage, have been studied.

Piergiovanni et al^5^ noted that lithoclast appeared to be the least traumatic among the lithotripters. Li et al^6^ has found higher incidence of ureteric stricture with holmium laser lithotripsy compared with PL. The thermal effect of lasers has been considered to cause an ablative effect on the mucosa, leading to fibrosis.^7^ These findings proposed that high temperature rise during the inappropriate use of the laser as one of the factors for stricture.

It is known that the threshold for causing thermal injury to cells is 43°C, and this threshold has been observed to be surpassed, even with low-power laser settings, in simulated models of renal calices and ureters.^8^ Hence, we decided to study the temperature rise between Ho:YAG and TFL lasers, to assess their safe use in laser lithotripsy procedures.

## Material and Methods

### Objective

To assess temperature variations using Ho-YAG and TFL lasers under different settings in a test tube at room temperature and when immersed in normal saline, which acts as a convection medium. 

All procedures performed in this study were in accordance with the ethical standards of the Institutional and National Research Committee. This study was approved by the Ethics Committee of Poona Medical Research Foundation (Approval no.: RHC/BIOPMRFIEC/2022/401, Date: 16th January 2023).

As our study is a laboratory experiment and does not involve patients or the animals, informed consent was not required.

### Experimental Design

**Figure 1.**
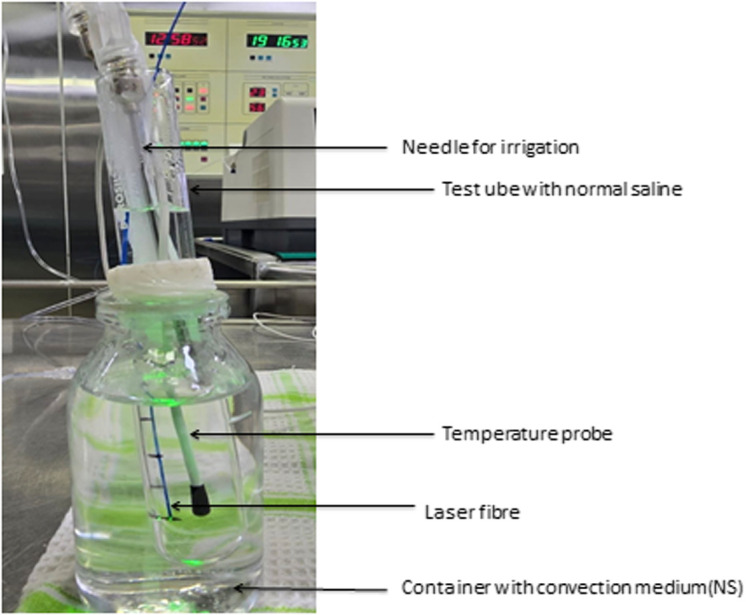
Test tube with irrigation needle, temperature probe, and laser fiber in a container with convection medium.

**Figure 2.**
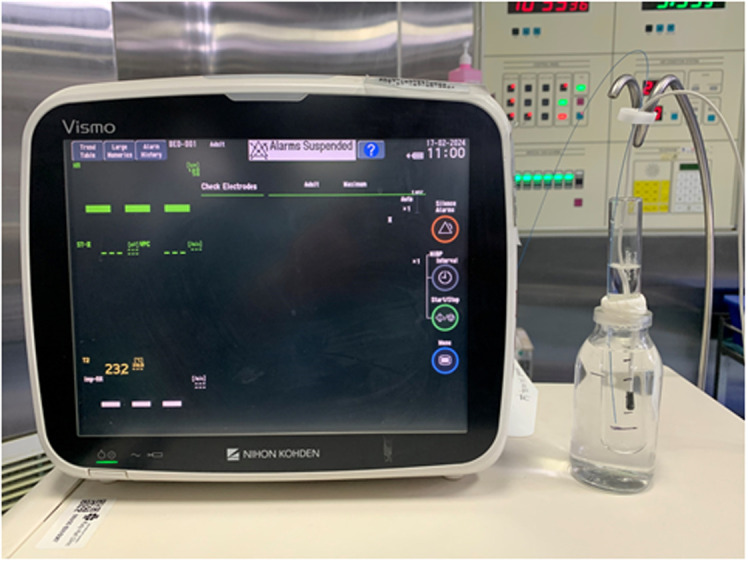
Monitor showing continuous temperature reading.

**Figure 3.**
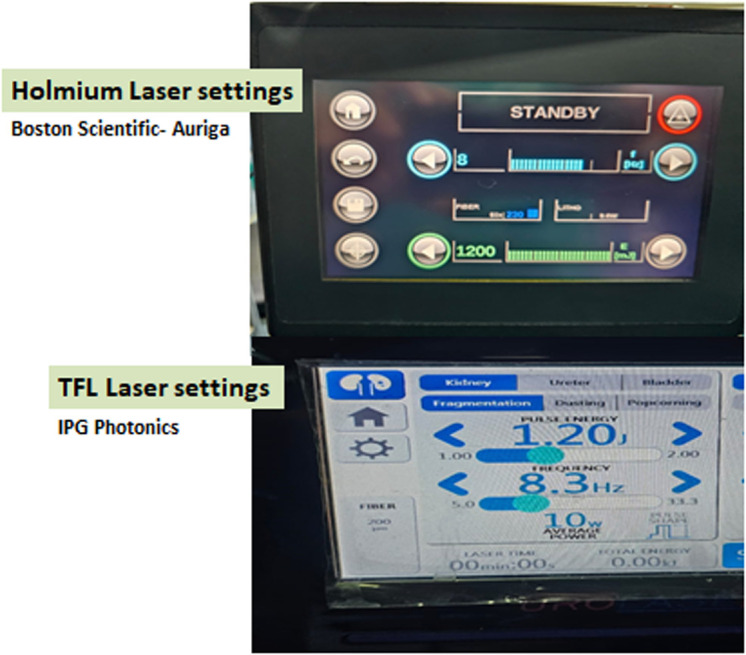
Laser machine monitor.

The experiment involved using a 10 mL test tube filled with normal saline. Room temperature was maintained at a constant 25°C, and the saline temperature matched the ambient room temperature, as shown in Figure 1. A standard PHILIPS esophageal temperature probe continuously recorded temperature on a cardiac monitor, as shown in Figure 2. Laser firing durations of 10, 20, and 30 seconds were used for both Ho-YAG and TFL lasers inside the test tube, with the temperature probe situated 1 cm away. A power of 10 and 20 W was applied to both lasers, varying energy and frequency settings, and temperature changes were recorded for each case. As shown in Figure 3, holmium laser was of a Boston Scientific-Auriga and the TFL laser was an IPG Photonics. A 200 µm laser fiber was used for 10 W power, and a 365 µm laser fiber was used for 20 W power.

Subsequently, the test tube was placed within an airtight transparent container filled with normal saline at room temperature, as described in Figure 1. The experiment was repeated, observing temperature changes with Ho-YAG and TFLs at 10 W and 20 W under different energy and frequency settings. Both these experiments were repeated 5 times each. Again, a similar experiment was repeated with irrigation at 10 mL/min and gravitational pressure of 60 cm H_2_O. This experimental setup aimed to elucidate the distinctions in temperature profiles between Ho-YAG and TFLs, considering various laser settings and the influence of an external environment such as immersion in normal saline.

### Statistical Analysis

Data were collected in a pre-designed proforma and later tabulated in a Microsoft Excel sheet. Data were analyzed using SPSS software version 20, (IBM SPSS Corp.; Armonk, NY, USA). The results on categorical data were shown as n (% of cases), and the data on continuous measurement were presented as mean ± standard deviation or median. The median and interquartile range were calculated for the final temperature. The non-parametric median test was used to compare holmium laser and TFL groups. A *P*-value ≤ .05 was considered statistically significant.

### Results

In a 10 cm^3^ test tube containing static normal saline at room temperature of 25°C, the temperature elevation caused by both holmium and TFL depended on the firing time while maintaining a constant power level. Tables 1A and 1B describe the temperature variations with holmium and TFL under different laser settings. For the holmium laser, with firing durations of 10, 20, and 30 seconds, the average temperatures increased to 35.1, 42.7, and 53.2°C, respectively. Meanwhile, TFL showed temperature increments of 38.3, 46.8, and 55.4°C under similar conditions. The power was consistently set at 10 W. Upon repeating the experiment with 20 W power, the temperature rise with both holmium and TFL was comparable to that with 10 W power as shown in Table 1B.

When the test tube was immersed in a normal saline container with a room temperature of 25°C, the average temperature rise was comparatively lower during laser firing durations of 10, 20, and 30 seconds, as shown in Tables 2A and 2B. For the holmium laser, temperatures reached 27, 30.8, and 39.4°C, while TFL demonstrated temperature increments of 27.8, 30.5, and 38.3°C, respectively. These findings highlight the influence of firing time and laser type on temperature dynamics, showing variations under different conditions, including immersion in normal saline, which acts as a convection medium.

As shown in Tables 3A and 3B, similar results were obtained when irrigation was used at 10 mL/min and gravitational pressure of 60 cm H_2_O. But, the maximum temperature was reached during longer lasing time and the time taken to reach baseline temperature was significantly lower with irrigation. When comparing the temperature rise between holmium and TFL, the differences in the rise of temperature were not statistically significant with irrigation and convection medium. 

## Discussion

Constructing a Ho:YAG laser involves a flash lamp, powered by a high-voltage supply. This light is diverted into a laser crystal rod containing holmium ions, which emit photons at 2100 nm. These photons oscillate between precisely aligned mirrors at the rod’s ends, forming a collimated beam in a reflective cavity. The continuous flash lamp pulsing sustains this process, creating a laser resonator. One mirror allows some radiation to escape, forming the output beam.^9,10^

Ureteroscopic holmium:YAG (Ho:YAG) laser lithotripsy is an effective and relatively safe surgical procedure for removing ureteral stones. Intraoperative complications are uncommon, occurring in only 3-5% of cases.^11^ Holmium:YAG lasers, known for their irregular and long-pulsed nature, primarily operate through a photothermal effect, minimizing the production of significant photoacoustic energy.^12^ Consequently, ureteral injuries associated with Ho:YAG lasers are likely a direct outcome of overheated tissue, which prompted the focus of our study.

Recently, TFL has been introduced and rapidly gained popularity due to its notable advantages over holmium. The TFL utilizes laser diodes as the energy source instead of a flash lamp. It produces a 1940 nm laser beam using a long thulium-ion-containing active fiber with a thin core diameter of 10-20 µm. This compact design enables direct coupling and delivery of the laser beam through another laser fiber to the target.^13^

In this study, we investigated the thermal aspect of the laser at different settings and compared Ho:YAG lasers and TFL. If used without irrigation, lasers can lead to high temperatures that damage renal tissue or the ureteric wall. Aldoukhi et al^14^ reported findings from an in-vivo porcine model study. They recorded higher temperatures with high energy or longer laser use. Inspection of the kidneys revealed gross pathological tissue coagulation and injury in their study.

A study by Hardy et al^15^ revealed that TFL has better results compared to holmium. Thulium fiber laser is twice as effective for fragmentation and four times more efficient in dusting compared to the Ho-YAG laser. Moreover, bubbles produced by TFL are four times smaller than those produced by holmium, resulting in less retropulsion by TFL.

However, literature suggests that TFL has a higher water absorption coefficient leading to high-temperature generation.^16^ Hardy et al^17^ performed temperature comparison between TFL and holmium in an in vitro study of a ureter model. The rise in temperature was seen more with TFL as compared with the holmium, especially at a higher frequency. Similarly, Molina et al^18^ also showed higher temperature rise with TFL as compared to holmium in a porcine model. Belle et al^19^ have demonstrated, in an in vitro silicone kidney-ureter model, that high-power lasers are associated with a risk of complications from thermal damage and recorded higher temperature rise with the TFL as compared with Ho:YAG laser at all tested laser settings. However, in our experiment, with the same lasing time and power, the temperature increase observed between holmium and TFL was similar, when irrigation was used.

Later, Taratkin et al^20^ performed the experiment to compare the temperature rise between TFL and holmium. There were no significant differences in temperature rise between 2 lasers, unlike the previous study. Æsøy et al^21^ studied temperature rise between TFL and holmium with three porcine kidneys with intact renal pelvis and proximal ureters. They recorded the temperature during continuous lasing time of 180 s at different power settings. They found that low power produced lesser temperature rise compared to high power both with TFL and holmium. In our study, we could compare the TFL laser at different power and observed that temperature rise was higher when high power was used. Instead of constant lasing time, we used different lasing time with both types of lasers to understand the importance of continuous firing a laser. We found that if power is kept constant, the temperature rise increases with an increase in lasing time from 10, 20, and 30 seconds, both with holmium and TFL lasers. 

The lasing time was directly proportional to the increase in temperature, even with irrigation. The difference was higher in static experiment. Tonyali et al,^22^ in their review of in vitro studies, highlighted that the key determinants of heat production are the laser settings and the duration of lasing. In our experimental setup as well, we found that longer firing times resulted in a more rapid rise in temperature while keeping power constant. We also observed that the time taken for the temperature to reach baseline is longer with lasing time. This is probably due to the higher temperature rise with the longer lasing time. These findings were comparable between holmium and TFL lasers.

We performed the experiment with two different power settings of 10 W and 20 W. Higher temperature rises were recorded with higher power settings. However, these differences were reduced with irrigation. Thus, total power was considered significant in temperature rise. Different modes of energy and frequency do not affect the temperature rise. Thus, use of dusting or the fragmentation during laser lithotripsy does not matter if the power is kept low.

The cooling time was significantly low when irrigation was used. This result shows that when inflow and outflow of irrigation fluid are properly maintained, the cumulative temperature rise is controlled. Hence, the rise in temperature to dangerous level is prevented. Wollin et al^11^ also discussed the role of saline irrigation in regulating temperatures during holmium:YAG (Ho:YAG) lithotripsy. They explained that as the irrigant flows over the tissue, stone, and surrounding urine, heat is transferred to the irrigant through convection. The study identified dangerously high temperatures across all laser settings when the irrigation flow was completely halted, with the lowest averaged maximum temperatures recorded at 47.1 and 47.2°C, which are cytotoxic. They also proposed that there exists a time–temperature relationship; for every 1°C beyond 43°C, the same amount of cell damage occurs half of the time. To illustrate, cells exposed to 45°C for 15 seconds would experience a similar level of cytotoxicity as cells exposed to 44°C for 30 seconds or 43°C for 1 minute.

Tonyali et al^22^ reviewed that if higher rates of irrigation fluid flow are used to counteract temperature rise, the rate of infection may increase. Pyelo-venous reflux, a condition where renal pelvic pressure surpasses 30-35 mm Hg, may occur.^23^ The absorption of chilled irrigation fluid into blood circulation through the surgical site can also lead to hypothermia.

In our experimental study, the factors that led to high temperature are laser settings with higher power, inadequate irrigation with poor exchange of fluid and longer lasing time. As seen in our study, temperatures to come back to baseline take a minimum of 15 seconds up to 22 seconds with convection medium. With continuous irrigation, it takes anywhere from 8 to 17 seconds, according to different laser settings. Hence, continuous lasing time and the time break between two lasing times are the most important points. It is also important to note that longer is the lasing time, longer is the time taken for the temperature after lasing to come back to normal.

Hence, if the laser machines have an inbuilt preset limit for lasing time of 15 seconds or maximum of 20 seconds, the temperature rise issue will automatically be taken care of. It is also proven that adequate irrigation is required during laser lithotripsy. The use of cold irrigation is not at all required and thus its side effects can be avoided.

This is an in vitro study and has its own limitations. First, this study did not include the artificial stone, and hence, the temperature change with regard to lasing stone can have minor changes in real scenarios. The stone formed in our body has various compositions, and artificial stone could not exactly mimic it; hence, the experiment was performed without stone. Second, even though commonly used laser settings are employed, the results may differ when applied to laser lithotripsy performed on patients because our study is in vitro.

The rise in temperature was almost equivalent to holmium and TFL. Hence, TFL can be safely used in laser lithotripsy under appropriate measures.

The increase in the temperature reaches a hazardous level without convection medium with continuous firing time of 20 seconds. In a container with convection medium of normal saline and with irrigation, temperature reaches close to hazardous limit with continuous firing time of 30 seconds. This was almost equivalent to holmium and TFL. Thus, to do lasing safely, continuous firing time should not go beyond 20 seconds, despite the type of lasers. Continuous irrigation is strongly recommended and urologist should confirm the irrigation flow during the surgery. Laser settings at high power should be used cautiously.

It is strongly recommended that the laser machine should have an inbuilt mechanism to decide the waiting time between laser firings as per the previous lasing time and power used.

## Figures and Tables

**Table 1A.** Temperature Assessment at Room Air at 10 W Power

	**Holmium (1.2 J × 8 Hz)**	**TFL (1.2 J × 8.3 Hz)**	***P***
**10 seconds**	35.1 (34.9-35.3)	38.3 (38.1-39.1)	<.05
**20 seconds**	42.7 (41.2-43.2)	46.8 (46.2-46.9)	<.05
**30 seconds**	53.2 (52.9-53.6)	55.4 (55.1-56.1)	1
	**Holmium (0.6 J × 18 Hz)**	**TFL (0.5 J × 20 Hz)**	***P***
**10 seconds**	34.7 (34.2-34.9)	38.1 (37.8-38.6)	<.05
**20 seconds**	42.8 (42.2-42.9)	47.1 (46.5-47.8)	<.05
**30 seconds**	53 (52.7-53.5)	55 (54.8-55.6)	<.05

*P *> .05, not significantly different.

**Table 1B.** Temperature Assessment at Room Air at 20 W Power

	**Holmium (1.8 J × 12 Hz)**	**TFL (1.5 J × 13.3 Hz)**	***P***
**10 seconds**	34.3 (33.9-34.6)	39 (38.5-39.1)	<.05
**20 seconds**	42.7 (42.4-42.9)	46.8 (46.7-47)	<.05
**30 seconds**	55 (54.8-55.2)	56.3 (56.2-56.5)	1
	**Holmium (0.5 J × 40 Hz)**	**TFL (0.5 J × 40 Hz)**	***P***
**10 seconds**	36 (35.6-36.3)	38.7 (38.3-38.9)	<.05
**20 seconds**	42.1 (41.4-42.5)	48 (47.4-48.3)	<.05
**30 seconds**	54.2 (54-54.5)	57.1 (56.9-57.4)	<.05

*P *> .05, not significantly different.

**Table 2A.** Temperature Assessment in Convection Medium (Normal Saline) at 10 W Power

	**Holmium (1.2 J × 8 Hz)**	**TFL (1.2 J × 8.3 Hz)**	***P***
**10 seconds**	27 (26.6-27.3)	27.8 (27.3-27.9)	.524
**20 seconds**	30.8 (30.5-31)	30.5 (30.4-30.7)	1
**30 seconds**	39.4 (39.1-39.6)	38.3 (38.2-38.5)	.444
	**Holmium (0.6 J × 18 Hz)**	**TFL (0.5 J × 20 Hz)**	***P***
**10 seconds**	27.2 (26.8-27.5)	28.1 (27.7-28.3)	.444
**20 seconds**	31.1 (30.4-31.5)	30.2 (29.6-30.5)	.524
**30 seconds**	39.1 (30.4-39.4)	38.2 (38-38.5)	.206

*P *> .05, not significantly different.

**Table 2B.** Temperature Assessment in Convection Medium (Normal Saline) at 20 W Power

	**Holmium (2.5 J × 8 Hz)**	**TFL (2.0 J × 10 Hz)**	***P***
**10 seconds**	27 (26.6-27.2)	26.9 (26.7-27.1)	1
**20 seconds**	29.2 (29.1-29.5)	30.2 (30-30.5)	1
**30 seconds**	38.9 (38.1-39.4)	39 (38.6-39.2)	1
	**Holmium (0.5 J × 40 Hz)**	**TFL (0.4 J × 50 Hz)**	***P***
**10 seconds**	27.2 (26.7-27.3)	27.8 (27.5-28.1)	.206
**20 seconds **	29.1 (28.6-29.4)	31 (30.5-31.4)	.167
**30 seconds**	37.1 (37-37.5)	37 (36.7-37.4)	1

*P *> .05, not significantly different.

**Table 3A.** Temperature Assessment with Irrigation (Normal Saline) at 10 W Power

	**Holmium (1.8 J × 6 Hz)**	**TFL (1.0 J × 10 Hz)**	***P***
**10 seconds**	30.2 (29.8-30.4)	30.8 (30.6-31.5)	.206
**20 seconds**	30 (29.9-30.3)	30.6 (30.4-30.9)	.524
**30 seconds**	29.1 (28.3-29.6)	28.8 (28.4-29)	1
	**Holmium (0.6 J × 18 Hz)**	**TFL (0.5 J × 20 Hz)**	***P***
**10 seconds**	29.8 (29.3-29.9)	30.4 (30.1-30.7)	.444
**20 seconds**	29.6 (29.1-29.9)	30.1 (29.6-30.5)	1
**30 seconds**	29.2 (29.1-29.6)	29.7 (29.4-30.2)	1

*P *> .05, not significantly different.

**Table 3B.** Temperature Assessment with Irrigation (Normal Saline) at 20 W Power

	**Holmium (1.8 J × 12 Hz)**	**TFL (1.5 J × 13.3Hz)**	***P***
**10 seconds**	28.9 (28.5-29.1)	29.5 (29.3-30.2)	.524
**20 seconds**	29.3 (29.2-29.6)	29 (28.8-29.3)	1
**30 seconds**	30.2 (29.4-30.7)	30.8 (30.4-31)	1
	**Holmium (0.5 J × 40 Hz)**	**TFL (0.5 J × 40 Hz)**	***P***
**10 seconds**	29.5 (29-29.6)	30.3 (30-30.6)	.444
**20 seconds**	29 (28.5-29.3)	28.1 (27.6-28.8)	1
**30 seconds**	30.5 (30.4-30.9)	29.8 (29.5-30.3)	1

*P *> .05, not significantly different.

## Data Availability

The data of this study is available upon request to the corresponding author.
